# Phenotypic variation, functional traits repeatability and core collection inference in *Synsepalum dulcificum* (Schumach & Thonn.) Daniell reveals the Dahomey Gap as a centre of diversity

**DOI:** 10.1038/s41598-020-76103-4

**Published:** 2020-11-11

**Authors:** Dèdéou A. Tchokponhoué, Enoch G. Achigan-Dako, Sognigbé N’Danikou, Daniel Nyadanu, Rémi Kahane, Jacob Houéto, Nicodème V. Fassinou Hotegni, Alfred O. Odindo, Julia Sibiya

**Affiliations:** 1 School of Agricultural, Earth and Environmental Sciences, University of KwaZulu-Natal, Scottsville, Private Bag X01, Pietermaritzburg, 3209 South Africa; 2 Laboratory of Genetics, Horticulture, and Seed Science (GBioS), School of Plant Sciences, University of Abomey-Calavi, 01 BP 526, Abomey-Calavi, Benin; 3 Cocoa Research Institute of Ghana (CRIG), P. O. Box 8, Akim Tafo, Ghana; 4 Research Unit HortSys, Department Persyst, CIRAD, Campus de Baillarguet, 34398 Montpellier Cedex 5, France; 5 World Vegetable Center, East and Southern Africa, PoBox 10, Duluti, Arusha, Tanzania

**Keywords:** Plant sciences, Ecology

## Abstract

The miracle plant *Synsepalum dulcificum* is a multipurpose natural sweetener and a promising West African orphan fruit shrub candidate for genetic improvement. Unfortunately, basic knowledge such as phenotypic variation and inheritance estimates required for implementing a breeding program are still lacking. A set of 203 accessions were sampled in two habitats from seven populations spread across the Dahomey Gap (DG) and the Upper Guinea forest (UG) in West Africa. The phenotypic diversity and allometric relationships among functional traits were analysed; the broad-sense heritability was estimated for fruit-traits, and a mini-core collection was developed in the species. Quantitative variation in tree- and fruit-traits was recorded, and multivariate analyses were performed to assess relationships among accessions, whereas heritability was estimated using the coefficient of repeatability. Tree-traits observed in *S. dulcificum* were more variable than fruit-traits. While habitat-type only affected tree-traits, the provenance population significantly affected both fruit- and tree-traits, with the UG populations outperforming the DG ones. Significant correlations were observed among fruit-traits on one hand, and among tree-traits on the other hand, whereas poor correlations were observed between tree- and fruit-traits. The multivariate analysis grouped accessions in three clusters. Promising individuals for high fruit mass and pulp-dense genotypes’ selection were identified within clusters. Repeatability estimates for fruit-traits ranged from 0.015 (edible ratio) to 0.88 (fruit mass). The Core Hunter algorithm enabled the extraction of 41 individuals as robust representatives of the initial set of 203 accessions, and the mapping of this core collection suggested Dahomey Gap as a centre of diversity of the species. These original findings offer opportunities, not only for the genetic improvement of *S. dulcificum,* but also for targeted ex-situ conservation in the species.

## Introduction

**Figure 1:**
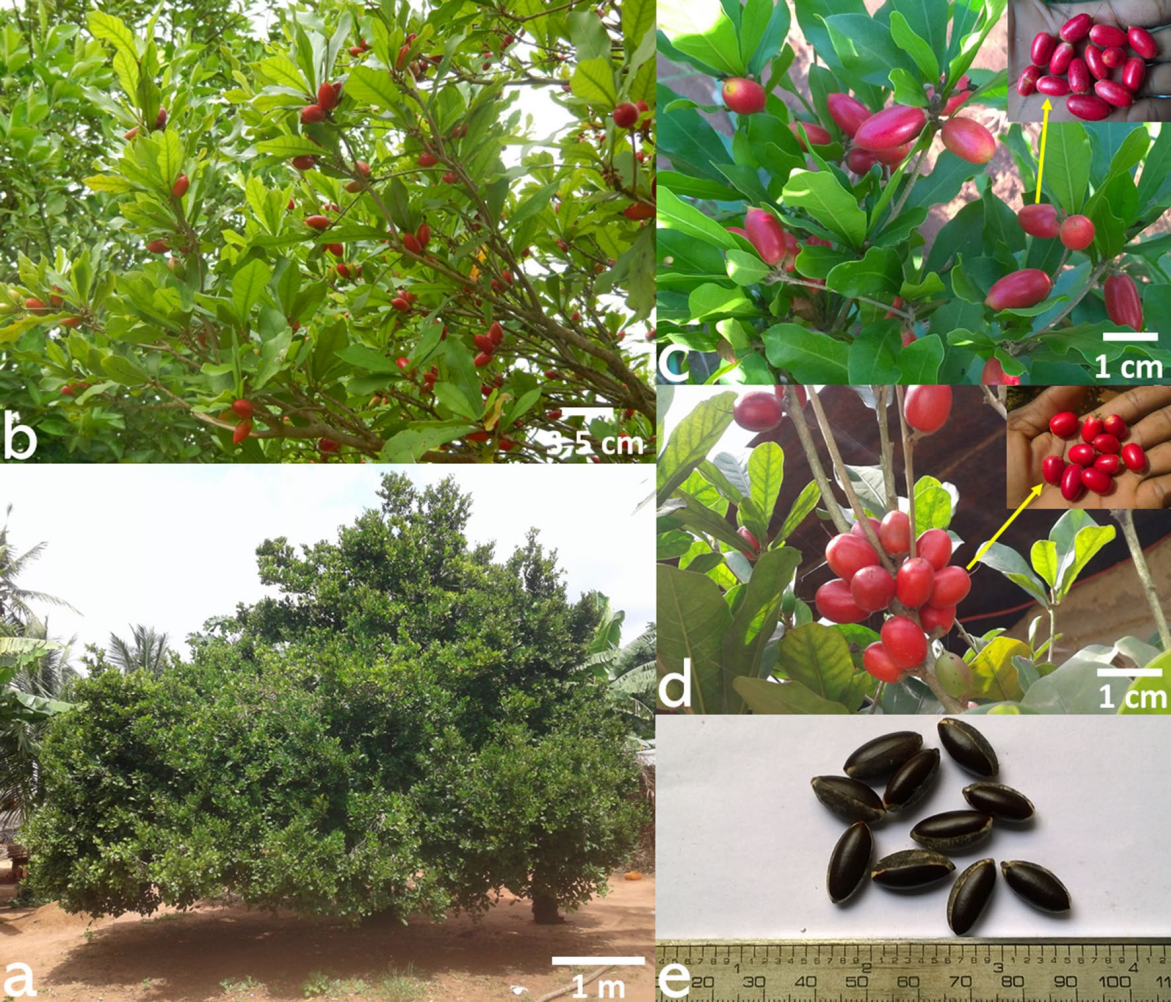
*Synsepalum dulcificum*, the studied species and some of its key organs. (**a**) A 110-year-old tree in home garden in Mission-Tove, Togo; (**b**) *S. dulcificum* branches bearing ripe fruits (miracle berries); (**c**) Oblong-shaped miracle berries; (**d**) Ovoid-shaped miracle berries; and (**e**) *S. dulcificum* seeds.

The miracle plant *Synsepalum dulcificum* (Schumach & Thonn.) Daniell [Syn. *Richardella dulcifica* (Schumach & Thonn.) Baehni] (Fig. 1a,b)—Sapotaceae—is a West African native shrub that produces a red fruit known as “miracle berry” (Fig. 1c,d). The berry is a unique natural source of miraculin^1^, a glycoprotein contained in the pulp that induces sweetness. *Synsepalum dulcificum* has numerous other applications ranging from traditional to modern uses^2^. Traditionally, all the non-edible parts of the species are involved in the treatment of many ailments including malaria, enuresis, coughing, and tooth decay among others^3,4^. Currently, the miracle berry is considered as a reliable alternative to synthetic sugar^5,6^ and helps to control obesity^7^ and diabetes^8–10^ in modern pharmaceuticals. It also serves as a food additive with its red skin and whitish pulp being, respectively, used for colouring and sweetening beverages and foods^11^. Applications of the species in cosmetics relate to the seed (Fig. 1e) oil used in hair breakage control and the improvement of hand and finger motor skills^12,13^. In West Africa, particularly in Benin, the miracle fruit is also sold on the open market and thus contributes to improving household livelihoods through income generation^3^.

Despite its growing market value (link1, link2), *S. dulcificum* is on the red list of the International Union for Conservation of Nature and classified as an endangered species in Benin^14^, with a risk of total depletion if no action is taken. Unfortunately, no conservation effort at either the local, regional and global scale has been so far reported in the species. Significant advances have been made in the development of farmer-friendly regeneration protocols^15^ and cultivation practices^16,17^ for production of *S. dulcificum* as a horticultural crop. However, foundation research for the genetic improvement and gene pool conservation of the species is still scanty. With an increasing interest in *S. dulcificum*^18,19^ worldwide, the economic potential of the shrub can be exploited if elite cultivars with improved traits are developed. However, the lack of basic knowledge on the genetics of the species is perceived as a bottleneck to the development and implementation of a relevant breeding program. For instance, the extent of genetic variation in *S. dulcificum* is elusive, and until now, there has been no study on the phenotypic diversity of the species. In their report on the molecular diversity of the species, Chibuzor et al*.*^20^ did not include any quantitative phenotypic variation. While ex-situ evaluation is a common approach for the phenotypic characterization of annual and biannual plant genetic resources^21^, in-situ evaluation is important in any preliminary study of the morphological variation in perennial species^22,23^. This has been shown in many fruit tree species including black raspberry (*Rubus occidentalis* L.)^24^, sumac (*Rhus coriaria* (L.) Kuntze)^25^, ber (*Ziziphus* spp.)^26^, wild almond (*Prunus scoparia* L.)^27^ and the African bush mangoes (*Irvingia* spp.)^28^, among others. This approach also provides primary information to shape a gene pool conservation strategy^29^, and sounds relevant when it comes to an orphan and threatened species such as the miracle plant.

Given that the fruit is the most valued and economically important organ in *S. dulcificum,* fruit and seed traits thus represent good candidates for genetic diversity studies and any selection. Predicting genetic gain for a target trait requires prior knowledge of its heritability^30^, and such information is currently unavailable in *S. dulcificum*. In perennial species, the coefficient of repeatability, which is the correlation between repeated measurements of the same individual over time and/or space is considered a reliable estimator of broad-sense heritability^31^. It has been used for fruit-traits heritability estimation in several tropical, subtropical and temperate tree species including mangabeira (*Hancornia speciose* Gomes)^32^, akebia [*Akebia trifoliate* (Thumb.) Koidz.]^33^, shea tree (*Vitellaria paradoxa* C.F. Gaertn.)^34^ and peach tree [*Prunus persica* (L.) Batch]^35^, among others. This metric appears relevant in the case of *S. dulcificum* to inform on the potential inheritance of fruit traits.

Core collection development emerged as an approach to optimize plant genetic resources conservation and utilization, but is still in its infancy for perennial and fruit tree species^36–38^. More importantly, no evidence exists for such resources in African indigenous underutilized fruit tree species, despite its potential to rationalize orphan fruit tree breeding process by helping to skirt the necessity to evaluate multi-traits on a large sample of individuals. Because *Synsepalum dulcificum* exhibits recalcitrant seeds^39^, cryopreservation appears to be the most adequate way for the maintenance of its genetic resources^40,41^. Unfortunately, cryopreservation is currently inexistent in West Africa. The only cost-effective alternative ways to conserve the species ex-situ is to establish it as living collections, whose size will be tremendously reduced, and the management much eased with the development of a core collection.

The current natural distribution area of *S. dulcificum* ranges from Ivory Coast to the Democratic Republic of Congo (link3). This area covers four major ecological regions of the African lowland rain forest including the Upper Guinea forest block (UG), the Dahomey Gap (DG), the Lower Guinea forest block (LG) and the Congolian forest block (CB)^42^. However, the species has been reported to originate from a complex of countries including Ghana, Togo and Benin^43–45^, which encompasses UG and DG.

Therefore, the objectives of the present study were to: (1) assess the phenotypic diversity of *S. dulcificum* among genetic resources from Benin, Togo and Ghana in West Africa; (2) estimate the heritability of some major fruit traits, and; (3) develop a core collection in the species. The overall goal of this study is to design and implement a relevant breeding program for the miracle plant in West Africa in order to sustain the utilization of the species. The study was guided by the following research questions: (1) what is the extent of quantitative variations in the miracle berry tree- and fruit-traits in its centre of origin and how are these traits affected by the ecological conditions?, (2) what is the clustering pattern among the miracle plant accessions?, (3) how heritable are the miracle berry traits? and (iv) what is the structure of the phenotype-based core collection in the species in the distribution area of West Africa including Benin, Togo and Ghana?

## Materials and methods

### Study area for sampling

**Figure 2:**
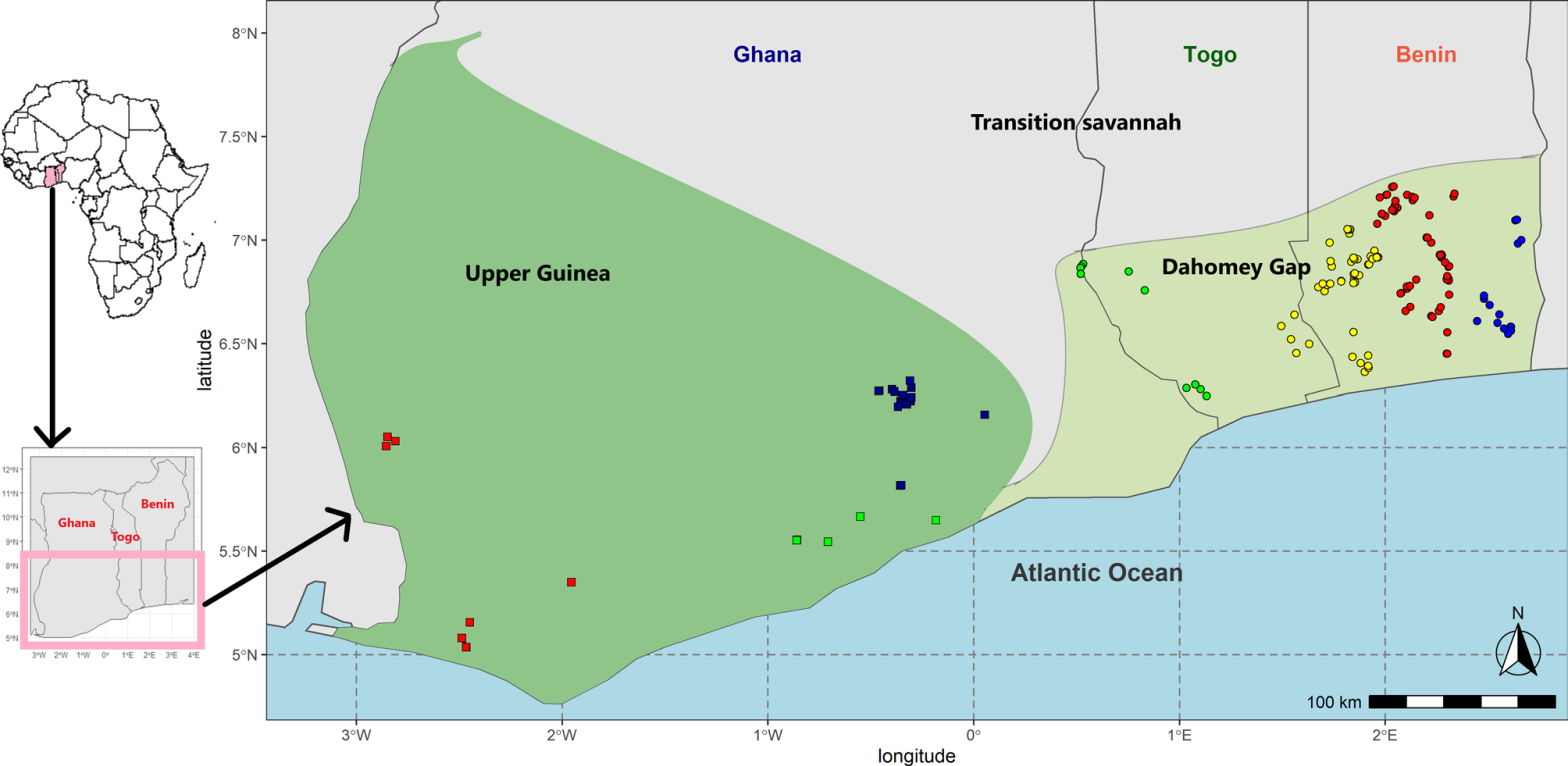
Study area showing the spatial distribution of the 203 accessions of *Synsepalum dulcificum* sampled in West Africa. Accessions are coloured according to the seven population sites under study. Square shapes: Upper Guinea forest block populations (filled red square: Western, filled green square: Central, filled blue square**:** Eastern); and Round shapes: Dahomey Gap populations (filled green circle: Volta, filled yellow circle: Mono, filled red circle: Zou, filled blue circle: Oueme). Map generated using the R environment (https://www.r-project.org/) (Version 3.6.2).

The data collection focused on the Upper Guinea forest block (UG) and the Dahomey Gap (DG) (Fig. 2) and was expected to capture maximum diversity. The DG is a savannah corridor disrupting the zonal West African rain forest. It was created during the late Holocene following an abrupt climatic change between 4500 and 3400 cal. years BP^46^. It is considered as a geographical barrier between UG demarcated at the West through the Volta river, and LG demarcated at the East through the Weme river^47^. The DG zone experiences 1000–1200 mm annual rainfall. The vegetation is a mixture of savannahs, gallery forests, and fragments of swamp forests, as compared to that of UG, dominated by rain forest with > 2000 mm annual rainfall^48^. The UG block corresponds to the rain forest belt extending from Sierra Leone to Ghana^42^. A total of seven collecting sites were investigated including four (Oueme, Zou, Mono and Volta) in DG and three (Eastern, Central and Western) in UG, giving rise to seven populations (Fig. 2). A population of *S. dulcificum* was defined as a set of accessions possibly interbreeding and randomly distributed in an agroforestry or tree-based production system within the same area or environment. Specifically, two distant populations of *S. dulcificum* (e.g. Mono and Volta, Eastern and Western) were separated by the minimum geographical distance of 50 km whereas two adjacent populations of *S. dulcificum* were separated by a maximum distance of 15 km coupled with the existence of a natural barrier such as a mountain (as observed between the adjacent populations Central and Eastern, in Ghana) or a river (as observed between the adjacent populations Zou and Oueme, and between the adjacent populations Mono and Zou, in Benin).

From September 2016 to August 2019, a total of 203 accessions (individual trees) of *S. dulcificum* were sampled from the seven populations, with the number of accessions sampled per population varying from 4 to 66 (Oueme: 19, Zou: 67, Mono: 67, Volta: 11, Eastern: 25 Central: 4, and Western: 10). The sampled accessions were all of reproductive age (i.e., having borne fruits at least once), and bearing fruits during the field sampling. The minimum distance of 100 m was observed between two sampled shrubs in order to reduce occurrence of closely related individuals.

### Data collection

For each accession, data on the habitat type, tree-traits, fruit-traits, and the tree age (where possible) were recorded. The habitat was recorded as either a home garden, which is here defined according to Niñez^49^ as a production system characterized by its proximity to dwellings, and over which the owner has a certain control on the target products; or as a farm, which is defined as an open agricultural production space, and usually farther away from the dwellings than a home garden. The tree age was estimated by the owner (the planter) where applicable; otherwise one of his closest relatives (e.g. brother, child or grandchild) gave the information.

Three tree-traits and six fruit-traits were measured per accession (Table 1). The tree-traits included: 1. Plant height (PlHgt), 2. Tree diameter at ground level (DiamGr), and 3. Tree crown diameter (CrDiam). The fruit traits included: 4. Fruit length (AvFL), 5. Fruit width (AvFW), 6. Fresh fruit mass (AvFM), 7. Seed mass (AvSM), 8. Edible ratio (EdRt) which is the pulp-to-fruit ratio, and 9. Fruit shape index (FrSh).

**Table 1:** Quantitative variation in *Synsepalum dulcificum* tree- and fruit-traits (n = sample size for the measurement, Min: Minimum, Max: Maximum, SD: Standard deviation; CV: Coefficient of variation).

Traits	Traits code	N	Min	Max	Mean	SD	CV (%)
**Tree-traits**							
Tree diameter at ground level (cm)	DiamGr	203	4.28	68.78	19.12	10.20	53.34
Tree height (m)	PlHgt	203	0.86	7.50	3.47	1.27	36.59
Tree crown diameter (m)	CrDiam	203	0.75	8.80	4.24	1.62	38.20
**Fruit-traits**							
Fruit length (mm)	AvFL	1015	13.04	26.41	18.86	1.82	9.65
Fruit width (mm)	AvFW	1015	7.39	18.30	10.59	1.28	12.08
Individual fruit mass (g)	AvFM	8875	0.43	2.03	1.09	0.28	25.68
Individual seed mass (g)	AvSM	8875	0.16	0.60	0.35	0.07	20.00
Edible ratio (%)	EdRt	8875	45.25	83.01	66.99	7.17	10.70
Fruit shape index	FrSh	1015	1.06	2.39	1.79	0.18	10.05

The plant height was measured from the soil surface to the tallest point of the shrub using a tape measure. The crown diameter was obtained by averaging the measures taken in the south-north and east–west directions. The diameter at ground level was obtained using the Eq. (1):

1 $$DiamGr = C_{Gr} /\pi$$

where DiamGr is the tree diameter at ground level, and C_Gr_ is the circumference at ground level measured with a tape measure.

The fruit-traits were recorded in-situ on 20–50 mature, fully ripe (red) and pests-free fruits that were randomly collected from the four sides (South, East, West and North) of each tree and bulked in replicates of 10 fruits each. A total of 8875 single fruits were assessed for this study.

The fresh mass of each replicate (FM_10_) was measured (± 0.01 g) using a portable electronic scale (OHAUS, model: PA512, Port Melbourne, Australia). The mean value of the replicates was then divided by 10 to obtain an average fruit mass (AvFM) of the accession. The same procedure was used to obtain the average seed mass (AvSM) of each accession after the fruits were depulped, while being kept in their replicate lot. The Edible Ratio (EdRt) was determined on each replicate, and the mean EdRt of an accession was the average value of all replicates of the accession. The EdRt was computed following the Eq. (2):

2 $$EdRt = \left( {FM_{10} - SM_{10} } \right)/FM_{10}$$

where FM_10_ is the fresh mass of 10 fruits, and SM_10_ is the fresh mass of the seeds of the same 10 fruits.

The fruit length and width were measured on five fruits randomly sampled from the different replicates per accession, using a digital Vernier calliper (± 0.01 mm). The mean values of all replicates per accession were used as the average fruit length (AvFL) and width (AvFW) of the accession. The fruit shape index (FrSh) of each accession was determined from five fruits following the Eq. (3):

3 $$FrSh = AvFL/AvFW$$

### Data analysis

All the analyses were performed using the R language Version 3.6.2^50^.

#### Tree morphology and fruit-trait variation

Descriptive statistics (minimum, maximum, mean, standard deviation, coefficient of variation) were computed to understand the overall variation in the species. The effect of the habitat-type on the tree height, diameter at ground level, crown diameter, fruit mass, fruit length, fruit width, fruit shape index and seed mass were assessed using a t-test, or a Mann Whitney’s test, where relevant. Likewise, the effect of the provenance population of accessions on the same variables was analysed using a type-II analysis of variance (ANOVA) to account for sample size imbalance among populations, or a Kruskal–Wallis test, where relevant. The edible ratio was assessed using a generalized linear model with a quasi-binomial error structure to account for overdispersion. Where the effect of the population was significant, a contrast analysis was employed to depict the influence of the ecological region (Upper Guinea vs Dahomey Gap) on the different parameters tested.

#### Relationship among tree morphology and fruit-traits

Relationships among tree- and fruit-traits were sought, and their significance were tested using Pearson’s or Spearman’s correlation tests, as appropriate. The correlation strength and significance were illustrated using the *chart.Correlation ()* function of the “PerfomanceAnalytics” package^51^. To depict the effect of the ecological region on the relationship among functional fruit-traits in the species, allometric regressions were used with sequential tests for slope equality and elevation shift between DG and UG accessions for various regression lines. This was done using the *sma ()* function of the “smatr” package^52^, whereas regression lines were drawn using the *ggPredict ()* function of the “ggiraphExtra” package^53^.

To assess the relationships among and between accessions and tree- and fruit-traits, a series of multivariate analyses were conducted. First, a Principal Component Analysis (PCA) was performed using the *PCA ()* function implemented in the “FactoMineR” package^54^ to retain the most meaningful components. Second, the clustering tendency in the dataset was tested using the Hopkins statistics “H” through the *hopkins ()* function implemented in the “clustertend” package^55^. A value of H close to 0.5 indicated that the dataset was not clusterable, whereas a value of H close to 0 indicated a clusterable dataset. In the latter case, a hierarchical clustering on the retained principal components (HCPC) was performed using the *HCPC ()* function of the “FactoMineR” package. The graphical outputs were visualized using the function *fviz_cluster ()* of the “factoextra” package^56^.

#### Estimation of repeatability

For each of the six fruit-traits, the repeatability estimation was conducted using the linear mixed effect models framework implemented in the “rptR” package^57^. To that, the provenance population was set as a fixed effect and the tree/accession as a random effect. The repeatability of the edible ratio was estimated using a generalized linear mixed-effect model with the *rptProportion ()* function whereas repeatability estimates of other fruit traits (fruit mass, seed mass, fruit length, fruit width and fruit shape index) were determined using a generalized linear mixed-effects model fitted by restricted maximum likelihood (REML) with the *rptGaussian ()* function. The interest in using the “rptR” package over the classical approach was mainly its ability to deal with non-gaussian data (e.g. the edible ratio in our case that is a proportion data). The significance of each repeatability was tested using the Likelihood Ratio Test (LRT), and the uncertainties (standard error: S.E. and Confidence Interval: C.I.) associated to the repeatability estimates were determined using a parametric bootstrapping (n = 1000 data samples).

#### Development and evaluation of the core collection

A Core Hunter phenotype data was developed using the *phenotype ()* function implemented in Core Hunter version 3 of the “Core Hunter” package^58^. Thereafter the *samplecore ()* function of the same package was applied to the Core Hunter data previously generated to develop the core set of accessions using the average-entry-to-nearest-entry distance optimization objective supported by the Gower distance measure. The quality of the core collection developed was evaluated by first computing the Coincidence Rate of range (CR%) (Eq. 4), the Variable Rate (VR%) (Eq. 5), the Variance Difference percentage (VD%) (Eq. 6), and the Mean Difference percentage (MD%) (Eq. 7) between the whole collection and the core collection, following Kim et al*.*^59^ and Hu et al*.*^60^:

4 $$CR\% = \frac{1}{m}\mathop \sum \limits_{i = 1}^{m} \frac{{R_{{C_{i} }} }}{{R_{{W_{i} }} }} \times 100$$

where ${\text{R}}_{{{\text{C}}_{{\text{i}}} }}$ is the range of the core collection for the trait i, and ${\text{R}}_{{{\text{W}}_{{\text{i}}} }}$ is the range of the whole collection for the trait i;

5 $$VR\% = \frac{1}{m}\mathop \sum \limits_{i = 1}^{m} \frac{{CV_{{C_{i} }} }}{{CV_{{W_{i} }} }} \times 100$$

where ${\text{CV}}_{{{\text{C}}_{{\text{i}}} }}$ is the coefficient of variation of the core collection for the trait i, and ${\text{CV}}_{{{\text{W}}_{{\text{i}}} }}$ is the coefficient of variation of the whole collection for the trait i;

6 $$VD\% = \frac{1}{m}\mathop \sum \limits_{i = 1}^{m} \frac{{\left| {V_{{W_{i} }} - V_{{C_{i} }} } \right|}}{{V_{{C_{i} }} }} \times 100$$

where ${\text{V}}_{{{\text{C}}_{{\text{i}}} }}$ is the variance of the core collection for the trait i, and ${\text{V}}_{{{\text{W}}_{{\text{i}}} }}$ is the variance of the whole collection for the trait i;

7 $$MD\% = \frac{1}{m}\mathop \sum \limits_{i = 1}^{m} \frac{{\left| {M_{{W_{i} }} - M_{{C_{i} }} } \right|}}{{M_{{C_{i} }} }} \times 100$$

where ${\text{M}}_{{{\text{C}}_{{\text{i}}} }}$ is the mean of the core collection for the trait i, and ${\text{M}}_{{{\text{W}}_{{\text{i}}} }} { }$ is the mean of the whole collection for the trait i.

Then, the existence of significant difference of phenotypic traits was tested between the whole collection and the core collection, using a t-test (for seed mass), a Wilcoxon’s test (for fruit mass, fruit length, fruit width, tree stem diameter, tree height, tree crown diameter and fruit shape index), or a generalized linear model with a quasi-binomial error distribution (for edible ratio).

Additional packages such as “ggplot2”^61^ (for basic graphics management), “sf”^62^, “rworldxtra”^63^ and “rnaturalearth”^64^ (for mapping and geo-referencing) were also used.

## Results

### Quantitative variation in tree- and fruit-traits

**Figure 3:**
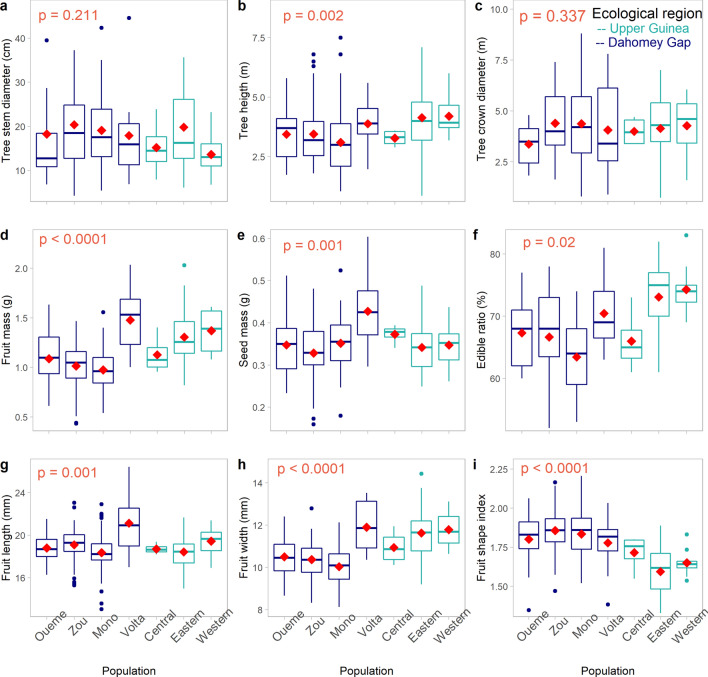
Quantitative variation of *Synsepalum dulcificum* tree- and fruit-traits across the seven sampled populations. Median values are in bold, red diamond shapes represent mean values, dots above and below boxplots are outliers, and lower and upper tails represent minimum and maximum values, respectively.

Tree-traits generally exhibited larger variation than fruit-traits (Table 1). The phenotypic coefficients of variation ranged from 36.59% (tree height) to 53.34% (tree diameter at ground level) for tree-traits, and from 9.65% (fruit length) to 25.68% (fruit mass) for fruit-traits. The effect of the population provenance was significant (*p* < 0.05) on all the assessed traits except on tree diameter (*p* = 0.211) and tree crown diameter (*p* = 0.337) (Fig. 3a–i). In particular, trees were taller in the Upper Guinea forest (UG) than in the Dahomey Gap (DG) (*p* = 0.001, Fig. 3b). Likewise, accessions from the UG produced heavier, longer and larger fruits with higher edible ratio than those from the DG (*p* < 0.0001, Fig. 3d–h), whereas fruit shape index (*p* < 0.0001, Fig. 3i) was greater in accessions from DG (oblong fruit) than from UG (round fruit). Among the screened populations, the Volta population produced bigger fruits and heavier seeds (*p* < 0.0001).

**Figure 4:**
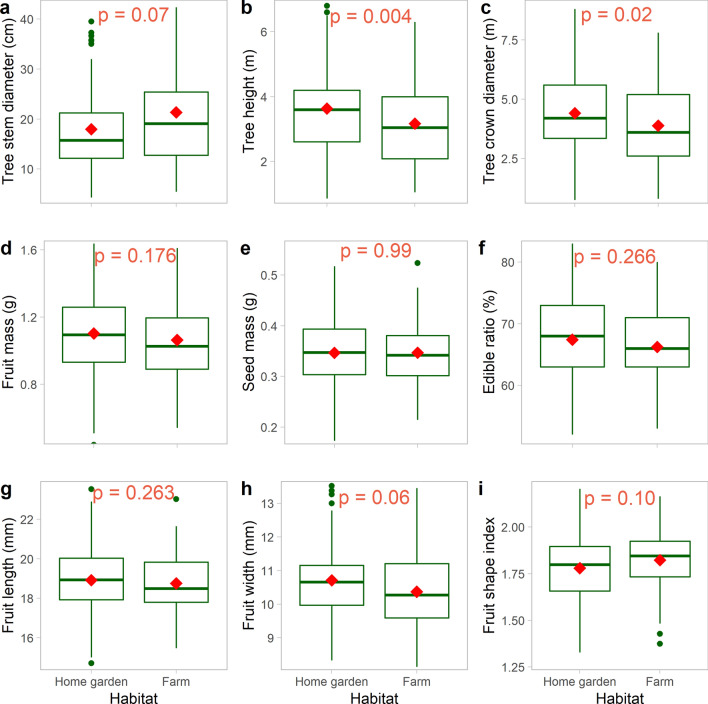
Quantitative variation of *Synsepalum dulcificum* tree- and fruit-traits across the habitat-types under study. Median values are in bold, red diamond shapes represent mean values, dots above and below boxplots are outliers, and lower and upper tails represent minimum and maximum values, respectively.

Contrary to the provenance population, habitat-type had lower effect on most studied traits (Fig. 4a–i), only significantly affecting tree-traits (tree height, *p* = 0.004, and crown diameter, *p* = 0.02), with relatively bigger shrubs in home gardens than in farms. Tree diameter at ground level (*p* = 0.07) was relatively greater in shrubs of farms than in those of home gardens. The age estimate of the studied trees ranged from 9 to 150 years and was on average 57.69 ± 2.04 years. No significant age difference was found between individuals on farms (57.75 ± 3.44 years) and those in home gardens (57.66 ± 2.56 years) (*p* = 0.56) (see Supplementary Fig. S1).

### Relationships among tree- and fruit-traits

**Figure 5:**
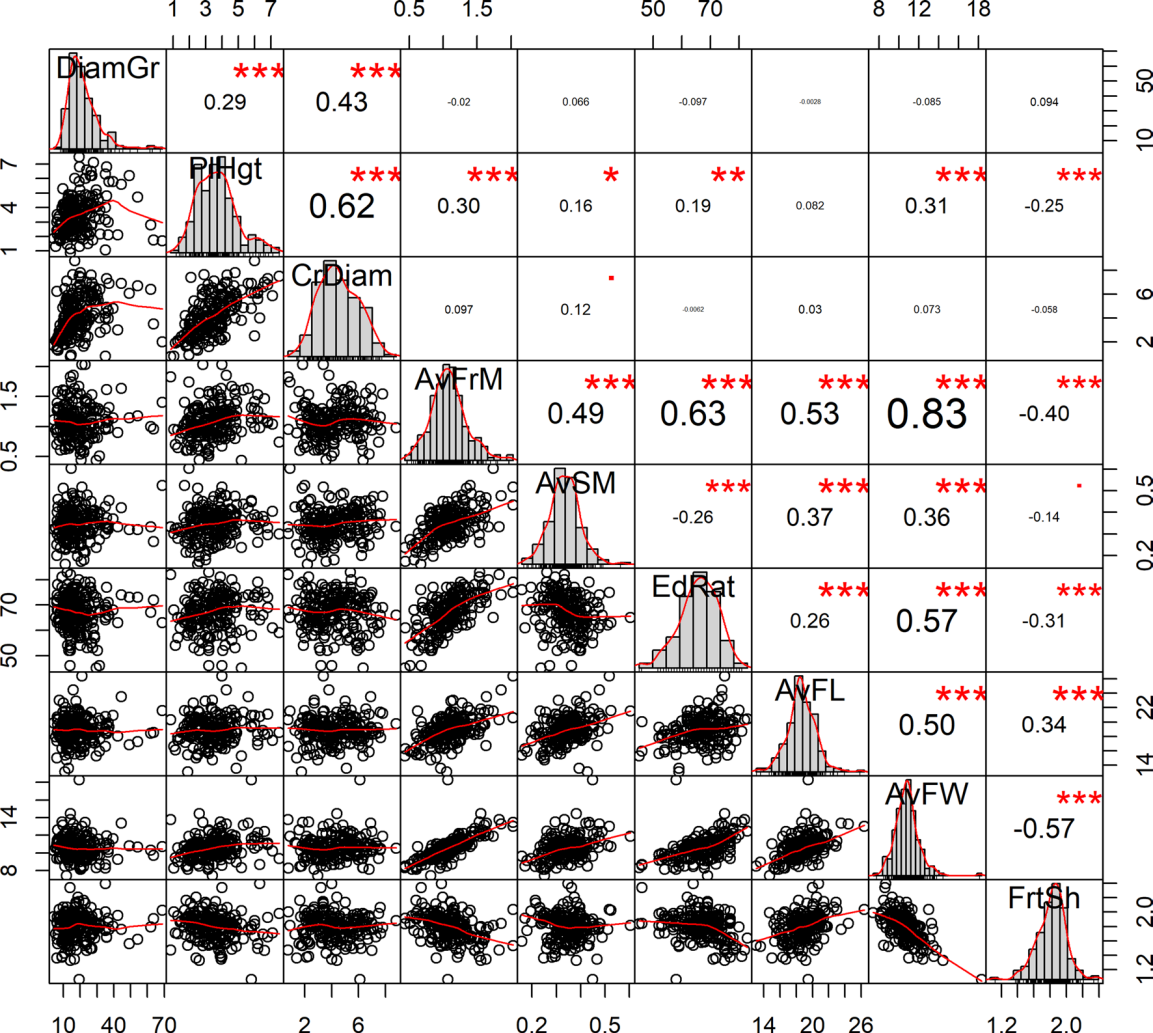
Mixed correlogram based on nine *Synsepalum dulcificum* tree- and fruit-traits (Traits list detailed in Table 1). On the main diagonal histograms are presented for each trait. Figures at the lower triangle present bivariate scatterplots, with a fitted line. Upper triangle contains correlation coefficients (r) with significance value from a correlation test. Magnification of r values is proportional to the correlation strength and magnification of the *p*-value value symbol is proportional to the significance level: *** for *p* < 0.001, ** for *p* < 0.01, * for *p* < 0.05, ^▀^ for *p* < 0.1.

**Figure 6:**
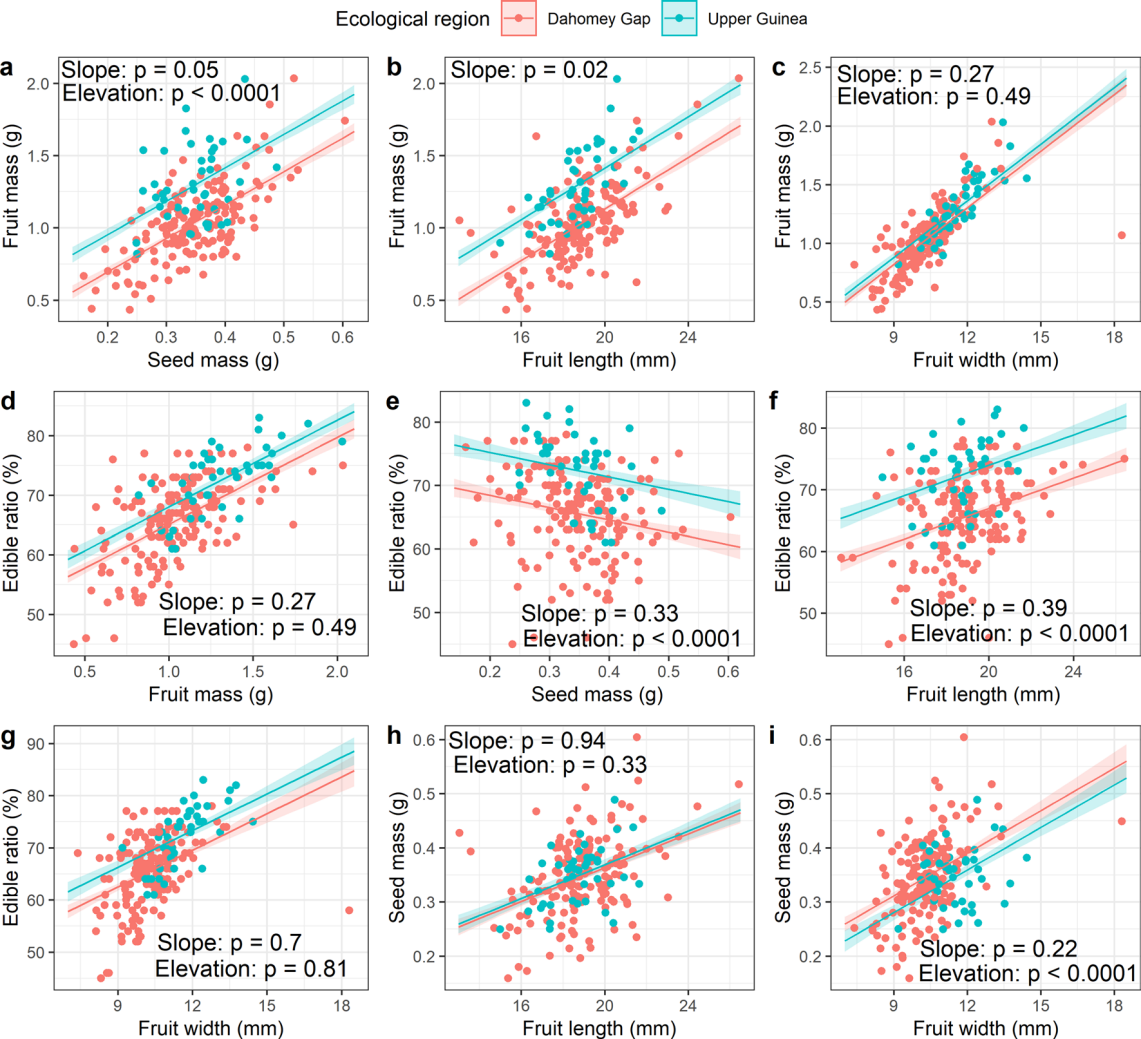
Allometric relationships among selected functional fruit-traits in *Synsepalum dulcificum* across the Dahomey Gap (in red) and the Upper Guinea forest (in blue) ecological regions. Slope: Test on difference between slopes; Elevation: Test on elevation shift in regression lines.

Coefficients of correlation ranged from − 0.00286 to 0.83 (Fig. 5). All tree-traits were positively and significantly correlated, and the strongest relationship was observed between tree height and crown diameter (r = 0.62, *p* < 0.001). The strongest relationship among fruit-traits was found between fruit mass and fruit width (r = 0.83, *p* < 0.001). However, fruit-traits were not uniformly correlated. Fruit mass was significantly correlated with all other fruit-traits (r ≥ 0.49, *p* < 0.01) except with fruit shape index (r = − 0.4), and fruit shape index was only correlated with fruit length (r = 0.34, *p* < 0.001). Edible ratio was positively correlated with fruit mass (r = 0.63, *p* < 0.001), but negatively with the seed mass (r = − 0.26, *p* < 0.001), meaning that larger fruits had shorter seed and denser pulp. Overall, there was a poor relationship between tree-traits and fruit-traits in *S. dulcificum*, and the highest correlation was observed between tree height and fruit width (r = 0.31, *p* < 0.01) (Fig. 5). Similarly, out of all the traits assessed, the tree age estimate was only correlated, and moderately to the tree diameter (r = 0.53, *p* < 0.001) and weakly to the tree crown diameter (r = 0.3*, p* < 0.001) (Supplementary Table S2). The ecological conditions seemed to influence the relationships observed between fruit-traits (Fig. 6a–i). For example, stronger correlations were observed between fruit mass and seed mass (Fig. 6a) on one hand and between fruit mass and fruit length (Fig. 6b) on the other hand for accessions from the UG compared with those from the DG. However, the correlation between fruit mass and fruit width was not influenced by ecological conditions (Fig. 6c). Similarly, correlations between edible ratio and fruit mass (Fig. 6d), edible ratio and fruit width (Fig. 6g), and between seed mass and fruit length (Fig. 6h) were not affected by ecological conditions. Nonetheless, a highly significant effect of the ecological region was noted on correlations between edible ratio and seed mass (Fig. 6e), edible ratio and fruit length (Fig. 6f) and between seed mass and fruit width (Fig. 6i). In this case, an increase in fruit width resulted in a greater seed mass gain in accessions from DG than those from UG, whereas a decrease in seed mass yielded a greater gain in edible ratio in accessions from UG than those from the DG. A greater correlation between edible ratio and fruit length was observed under UG ecological conditions than under DG conditions.

### Relationships among accessions of *S. dulcificum*

**Figure 7:**
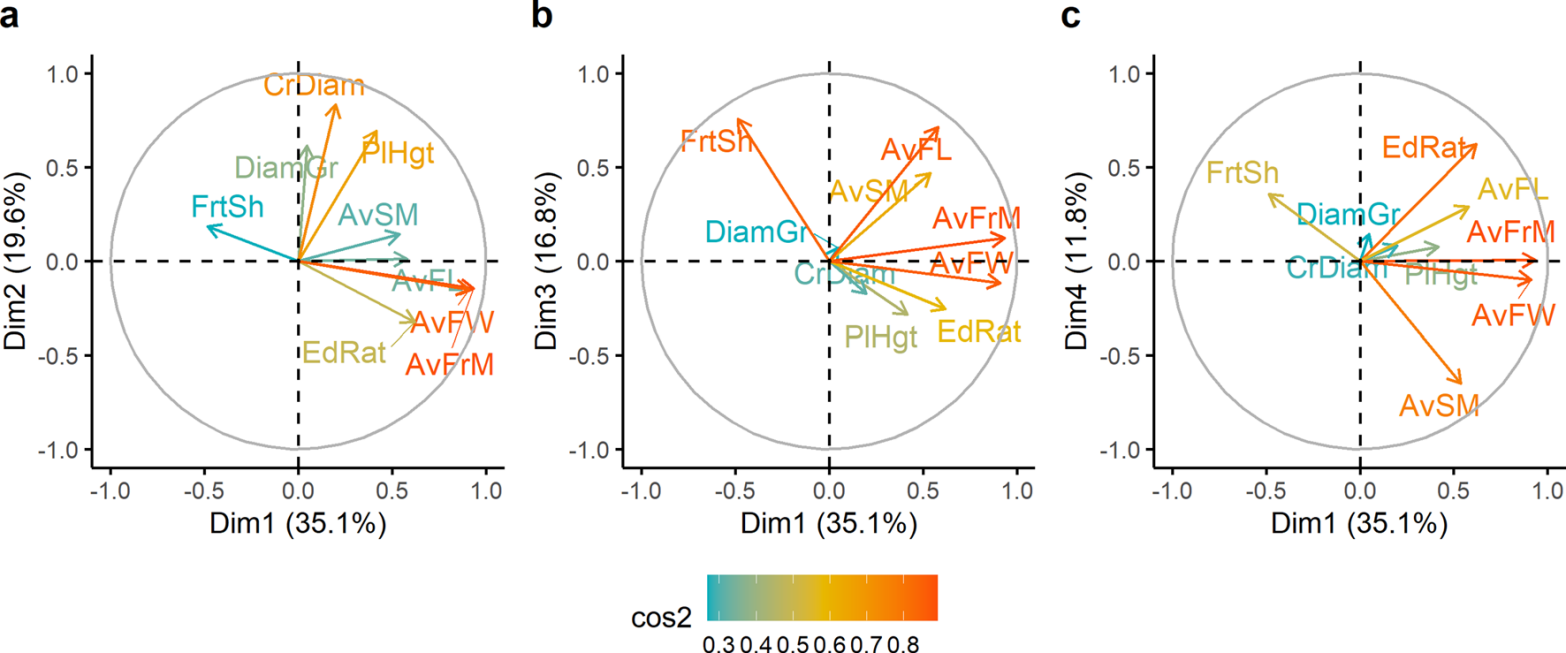
Correlation circle indicating *Synsepalum dulcificum* tree- and fruit-traits projection on the first two components: (**a**); first and third components: (**b**); and first and fourth components: (**c**) (traits list is detailed in Table 1).

From the principal component analysis, it emerged that the first four components explained a significant proportion (83.3%) of the total variance (Fig. 7a–c) and these were retained for further analyses. The most correlated traits to the first component (35.1% of the total variability) were fruit mass (r = 0.93, *p* < 0.0001), fruit width (r = 0.91, *p* < 0.0001) and edible ratio (r = 0.61, *p* < 0.0001). This first component is that of the “fruit size”. Similarly, the crown diameter (r = 0.83, *p* < 0.0001), tree height (r = 0.69, *p* < 0.0001) and tree diameter (r = 0.61, *p* < 0.0001) were the most correlated to the second component, which accounted for 19.6% of the total variability. This component is that of “tree-shape”. The fruit shape index (r = 0.756, *p* < 0.001) and fruit length (r = 0.71, *p* < 0.001) were the most represented variables on the third component “the fruit shape component” that accounted for 16.8% of the total variability. Though the component 4 accounted for 11.8% of the total variance, none of the variables were found to significantly correlate to it.

**Figure 8:**
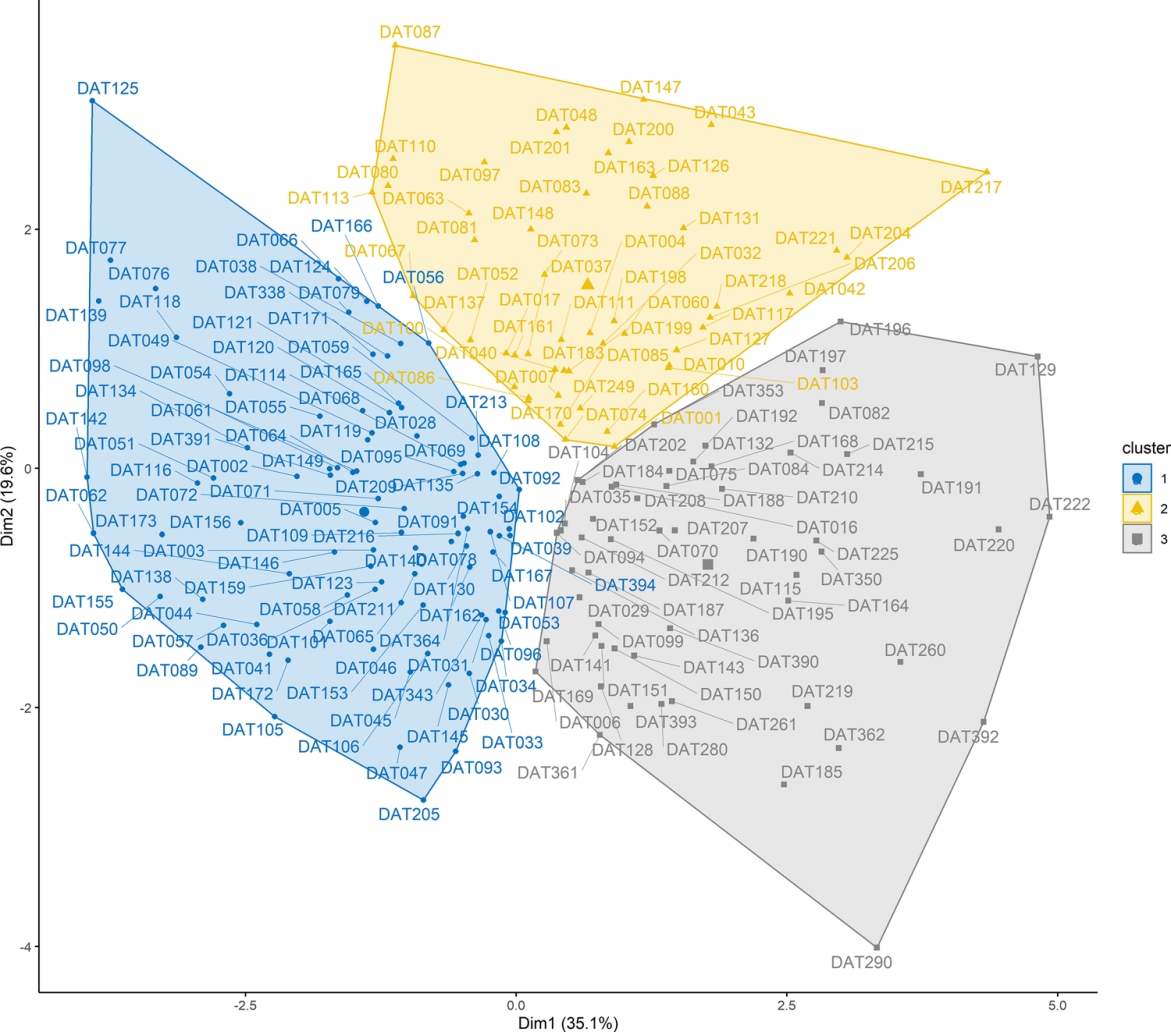
Factor map showing *Synsepalum dulcificum* accessions grouping in clusters.

The Hopkins statistics computed on the data was H = 0.23 (< 0.5), indicating the existence of significant clusters in the dataset. The hierarchical clustering on the first four components produced three clusters (Fig. 8). Clusters 1, 2 and 3 were made up of 46.8%, 26.5% and 26.7% of the total number of accessions, respectively and a χ^2^ test on the clustering structure indicated a significant representation of accessions from the Upper Guinea in cluster 3 (*p* = 0.001) (see Supplementary Fig. S3). The most determinant quantitative characteristics of each cluster are presented in Table 2.

**Table 2:** Phenotypic differentiation among *S. dulcificum* accession clusters.

Quantitative traits	Clusters		
	1	2	3
**Tree-traits**			
DiamGr	17.14 ± 0.88	**26.70 ± 12.21**	15.42 ± 6.21
PlHgt	2.81 ± 0.09	**4.59 ± 1.30**	NA
CrDiam	3.60 ± 0.12	**5.97 ± 1.20**	3.68 ± 1.36
**Fruit-traits**			
AvFL	17.99 ± 0.16	**19.45 ± 1.42**	**19.79 ± 1.78**
AvFW	9.76 ± 0.07	**NA**	**11.89 ± 1.21**
AvFM	0.88 ± 0.02	**NA**	**1.37 ± 0.22**
AvSM	0.31 ± 0.01	**0.37 ± 0.05**	**0.37 ± 0.06**
EdRt	64.01 ± 0.69	**NA**	**72.32 ± 5.34**
FrSh	**1.84** ± **0.02**	**NA**	1.67 ± 0.19

Figures in bold indicate values that are significantly greater than the overall means, while the rest of the figures indicate values that are significantly lower than the overall means for all accessions. NA: quantitative trait not significantly describing the cluster.

Cluster 1 was significantly characterized by all the nine traits studied, and mainly encompassed the least performing accessions observed in the study (Table 2). These accessions were of weak architecture, producing poor standard fruits that are markedly oblong in shape. Cluster 2 was significantly described by fruit length and seed mass in addition to tree traits (Table 2). Accessions in this cluster had a strong tree architecture and a high seed mass. Cluster 3 was characterized by individuals with a weak tree architecture, but with the highest performing fruit-traits. In part, accessions of cluster 3 had the highest fruit mass, fruit width, edible ratio, and seed mass (Table 2) and are the elite trees in this study.

### Fruit traits repeatability in *S. dulcificum*

Repeatability coefficients for the six fruit traits under study are presented in Table 3. All the repeatability estimates were highly significant (*p* < 0.0001) with most traits exhibiting high repeatability. Thus, the trait with the highest potential broad-sense heritability based on the repeatability estimates was fruit mass, whereas the one with the least broad-sense heritability was the edible ratio.

**Table 3:** Estimation of repeatability (adjusted) coefficient for six fruit-traits in *S. dulcificum* (*S.E.* standard error).

Fruit-traits	Repeatability (r_adj_)	Uncertainty^a^		Likelihood ratio test
		S.E	Confidence interval	
AvFL	0.745	0.023	[0.700, 0.786]	< 0.0001
AvFW	0.666	0.028	[0.608, 0.717]	< 0.0001
AvFrM	0.883	0.013	[0.855, 0.906]	< 0.0001
AvSM	0.821	0.019	[0.784, 0.857]	< 0.0001
EdRt	0.015	0.002	[0.010, 0.018]	< 0.0001
FrSh	0.580	0.030	[0.527, 0.647]	< 0.0001

^a^Determined using 1000 parametric bootstraps.

### Mapping the core collection of *S. dulcificum*

**Figure 9:**
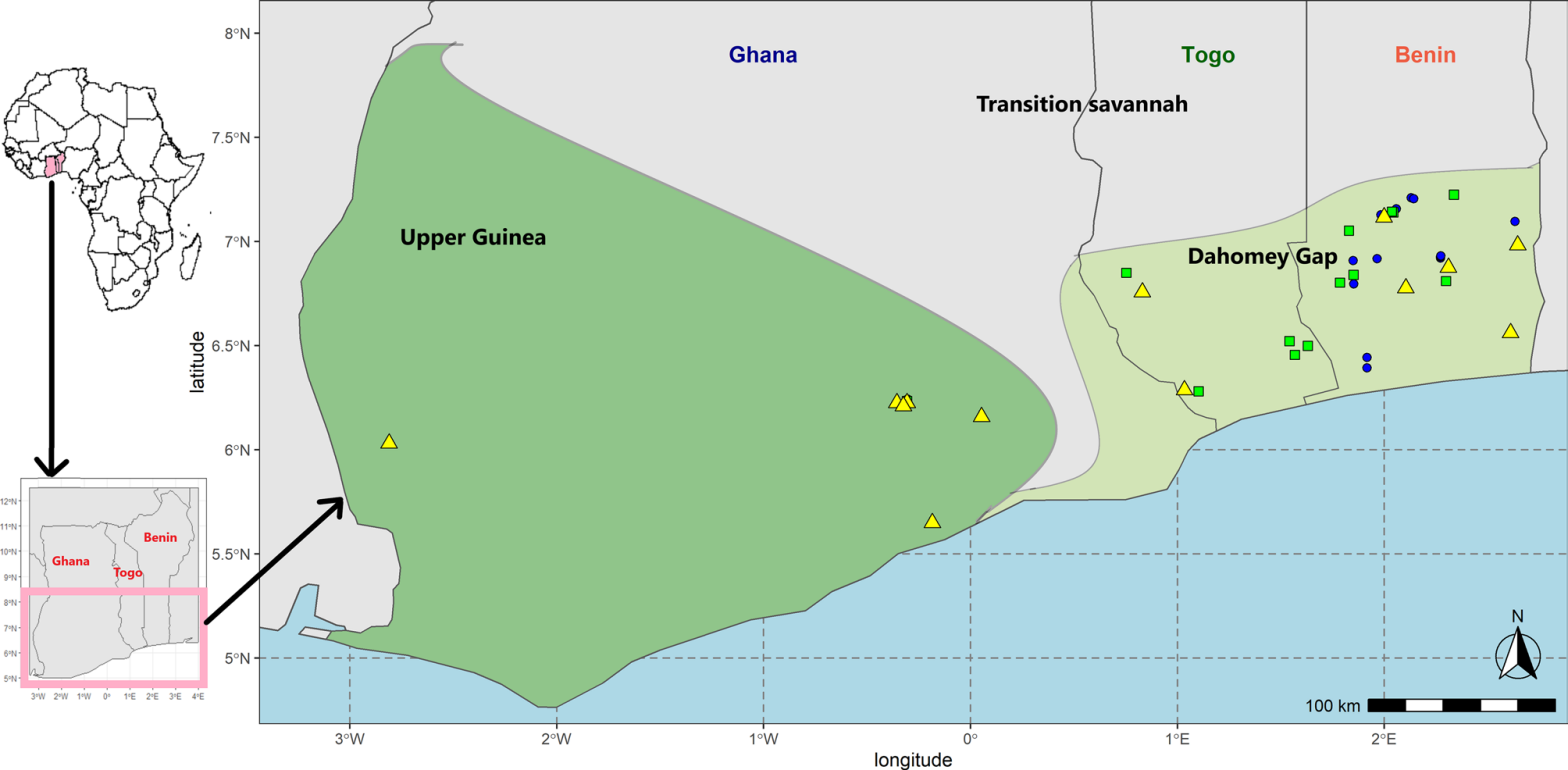
Spatial distribution of the core set (41 accessions: 8 accessions for UG and 33 for DG) extracted from 203 accessions of *Synsepalum dulcificum* in West Africa. Accessions are coloured following their belonging to the three clusters identified in this study: blue filled circle: Cluster 1 (least performant accessions with regards to the evaluated traits); green filled square: Cluster 2 (elite accessions for tree-traits); yellow filled triangle: Cluster 3 (potential elite accessions for fruit-traits). Map generated using the R environment (https://www.r-project.org/) (Version 3.6.2).

Based on the phenotypic data from the 203 accessions evaluated, the Core Hunter algorithm returned as core collection a total of 41 accessions. The spatial distribution of these 41 accessions is presented in Fig. 9. This core set is made up of eight accessions from the Upper Guinea forest and 33 from the Dahomey Gap. The repartition of the core set accessions in the three clusters obtained above indicated that Clusters 1, 2 and 3 were represented by 15, 13 and 13 accessions, respectively, with the Upper Guinea ecological region having mainly accessions of Cluster 3 against the Dahomey Gap region encompassing accessions from all the three clusters.

The coincidence rate of range (CR) per trait varied from 87.57 to 100% for an overall CR of 97.6% (Table 4). Similarly, all the variable rates (VR) per trait were higher than 100%, whereas the variance difference percentage between the whole collection and the core collection was 46.95%. More captivatingly, the difference between the whole collection and the core collection was not significant for any of the nine phenotypic traits evaluated (0.26 < *p* < 0.99). This observation was supported by the low mean difference percentage between the core and the whole collection, which was 3.23% (Table 4).

**Table 4:** Quality attributes of the core collection (Core) extracted form the whole collection (Whole).

Traits	Range		CRi (%)	CV		VRi (%)	Mean		MDi (%)	Variance		VDi (%)	Means comparison (whole vs core)
	Whole	Core		Whole	Core		Whole	Core		Whole	Core		
DiamGr	64.50	62.68	97.17	53.34	64.05	120.07	19.12	22.33	14.37	104.17	204.63	49.08	*p* = 0.26
PlHgt	6.64	6.64	100.00	36.59	45.88	125.38	3.47	3.70	6.21	1.613	2.87	43.90	*p* = 0.61
CrDiam	8.05	7.05	87.57	38.20	47.92	125.44	4.23	4.23	0.00	2.61	4.11	36.37	*p* = 0.99
AvFL	13.37	12.83	95.96	9.65	14.06	145.69	18.86	18.95	0.47	3.31	7.10	53.35	*p* = 0.89
AvFW	10.91	10.91	100.00	12.08	18.58	153.80	10.59	10.82	2.12	1.64	4.04	59.46	*p* = 0.52
AvFM	1.60	1.60	100.00	25.68	32.06	124.84	1.09	1.14	4.38	0.078	0.13	41.66	*p* = 0.43
AvSM	0.44	0.43	97.72	20.00	27.07	135.35	0.35	0.35	0.00	0.004	0.008	46.70	*p* = 0.82
EdRt	37.76	37.76	100.00	10.70	13.53	126.44	66.99	67.66	0.99	0.59	0.83	38.67	*p* = 0.44
FrSh	1.33	1.33	100.00	10.05	15.07	149.95	1.79	1.78	0.56	0.03	0.07	53.42	*p* = 0.97
			97.60			134.10			3.23			46.95	–

*CV* coefficient of variation, *Cri* coincidance range for the trait I, *VRi* variable range for the trait I, *VDi* variance difference for the trait I, *MDi* mean difference percentage for the trait i.

## Discussion

The study reports for the first-time, the phenotypic variation, heritability estimates for functional fruit traits and the development of a core collection for *S. dulcificum* in West Africa.

The miracle plant *S. dulcificum* is known to be an endangered species in Benin where it has been mostly reported in swamp forests^14^. However, from this current study, although not a purely ecological one, it is worth noting that no accession was found in swamp forest in Benin, but rather in farms and mostly in home gardens. In Togo, another portion of the Dahomey Gap, the species was notably absent from forests, especially from sacred forests where it had a rarity index of 0.98. This trend suggests that the predominant habitat of the species in the Dahomey Gap might have shifted from the natural environment to human-close environment such as home gardens, probably due to anthropogenic pressures such as land clearing for agricultural expansion. This seemed to align with the idea of home gardens as a biodiversity hotspot for endangered species^65^. In the Upper Guinea forest, the current absence of the species from forests might not only illustrate an in-situ anthropogenic pressure on the resource, but could also reflect a land conversion scenario in which forests were degraded in favour of tree plantation systems. This hypothesis is supported by previous reports indicating that the Upper Guinea forest has been experiencing serious degradations, which have already resulted in the loss of nearly 80% of the original forest cover^66^. Almost all the farms in which the species was recorded in the Upper Guinea forest zone were characterized by a tree plantation system where *S. dulcificum* was associated with cocoa (*Theobroma cacao* L.), rubber tree [*Hevea brasiliensis* (Willd. ex A.Juss.) Müll.Arg*.*], orange tree (*Citrus* spp) or *Musa* spp.

To date, no information existed on the variation of quantitative traits in *S. dulcificum*. In this study, using the populations sampled from the species’ centre of origin, trends of traits variations were similar to those observed in sister Sapotaceae species such as *V. paradoxa*^34,67^ from tropical Africa, and also *Argania spinosa* (L.) Skeels^68^ from Northern Africa. However, the magnitude of the variation observed in *S. dulcificum* is greater than in *V. paradoxa* and *A. spinosa* for tree-traits and lower in *S dulcificum* than in *V. paradoxa* and *A. spinosa* for fruit-traits. Considering tree-traits variation for instance, the difference in magnitude between *V. paradoxa* and *S. dulcificum* might be attributed to the degree of heterogeneity of the habitats of each species. Fundamentally, *V. paradoxa* is found in similar habitat-types (i.e. wild and parkland), whereas *S. dulcificum* is found in more diversified habitats including farms and home gardens where it undergoes different management intensities. In home gardens, *S. dulcificum* benefits from management practices such as watering and occasional fertilization compared to farms where there is no special management provided to the species. This is likely to increase the magnitude of the differences observed in tree-traits between these two habitats. Information on tree age is rarely reported in studies tackling phenotypic diversity in perennial fruit trees^69–71^. In this study, the age estimates for *S. dulcificum* confirmed the prominence of long-living species in the Sapotaceae family^72^. The absence of difference of age between home garden trees and those found on farms ruled out the hypothesis that tree age is a driver of tree-traits variation between these two habitats in *S. dulcificum*. This is furthermore supported by the overall poor correlation between tree age estimate and tree-traits.

This study provided for the first time information on *S. dulcificum* tree and crown diameter variation and expanded knowledge on the height growth potential of the species. *Synsepalum dulcificum* is a long-living microphanerophyte^14^ whose maximum height was reported to be around four meters^2^. However, in favourable conditions this height may reach up to seven meters and the crown diameter up to eleven meters. Such phenotypes are found mainly in home gardens since individuals on farms are prone to high human pressures including harvesting of leaves and branches as well as debarking of stem for medicinal purposes^3^, thus impeding their normal growth.

Ayensu^73^ successfully described the anatomy and morphology of *S. dulcificum* but made no mention of the fruit and seed morphometry. In an attempt to fill this gap, Lim^74^ in his botanical description of the species indicated the miracle fruit being roughly 1.0 cm wide for a length ranging from 2.0 to 2.5 cm. Nevertheless, this current study revealed greater variations for both fruit length (1.30–2.64 cm) and fruit width (0.73–1.83 cm). Given the significant effect the provenance population exhibited on fruit-traits in this study, differences with Lim’s^74^ findings could be attributed to environmental variation. On the other hand, a difference in sampling effort coupled with potential genetic variation among the individuals measured might also explain the differences observed in the reported fruit width and length. While Lim^74^ did not indicate the number of fruits used to compute the statistics, in this study nearly 8875 fruits were measured.

Due to the increasing interest in *S. dulcificum,* this study purposively targeted the fruit and its components. For instance, while the edible ratio is of great interest in medicinal, pharmaceutical, food and beverages industries^19^, the seed mass is of interest in pharmaceutical and cosmetics^12,13,75^. Consequently, knowledge of the tree-to tree or population-to population variation for these specific traits would be useful for elite tree selection by the breeders and decision making for investment by stakeholders. This study reported for the first time the extent of the edible ratio and seed mass variation in the miracle berry and showed the influence of the ecological conditions on the fruit traits in general. An average edible ratio of roughly 67% was observed in *S. dulcificum,* which is one of the highest when compared to other tropical Africa indigenous fruit species such as *V. paradoxa* (61.33–62.0% of edible ratio)^34,67^, *Balanites aegyptica* (L.) Delile. (44.9–50.65%)^76^, and subtropical species such as *A. trifoliata* (23.52–27.63%)^77^. More importantly, under favourable conditions, the edible ratio in *S. dulcificum* can increase up to 72% as observed for the set of individuals in the Upper Guinea forest block, a region characterized by higher rainfall and less sunlight exposure. This region was also favourable for heavier fruit production than the Dahomey Gap, which was characterized by drier conditions with savannah as main vegetation type. This strengthened arguments in favour of the previously highlighted beneficial effect of watering as well as the detrimental effect of sunlight exposure on *S. dulcificum* growth, reproductive and fruiting perfomances^16,17^. The influence of climatic conditions (e.g. rainfall) on fruit traits in perennial species was also previously reported in *Afzelia Africana* Smith^78^ in which individuals in wet conditions performed better than those in dry conditions.

The poor correlation observed in this study between *S. dulcificum* tree-traits and fruit-traits is in line with findings in other Africa indigenous fruit trees species (e.g. *V. paradoxa*^34,67^, *Sclerocaria birrea* (A. Rich) Hocchst.^79^), and suggests that tree architecture cannot be used to predict fruiting performance in adult individuals of *S. dulcificum*. Depending on the habitat, different pressures may interfere with the standard species growth, thus changing the trajectory of the relationship between tree morphology traits and any other group of traits. On the contrary, the significant and positive correlation between fruit mass and fruit width, and between fruit mass and the edible ratio, combined with the negative correlation between the edible ratio and seed mass are of significant interest for the development of a selection strategy in breeding of improved varieties of *S. dulcificum*. Such associations make it possible to simultaneously improve the fruit size and the edible ratio while improving the fruit mass. This could translate into an increased benefit and profitability for the food and beverages industries, which are more interested in the edible part of the fruit. On the other hand, it might be difficult to effectively select for seed mass using the fruit mass to benefit the cosmetics industry, thus a direct selection for high seed mass yielding genotypes would be required. Selection of dual-purpose miracle berry genotypes (e.g. for simultaneous use in both cosmetics and food and beverages industries) could be explored by developing a selection index that assigns weights of economic value for each trait (seed mass and edible ratio)^80^. More importantly, the influence of the ecological conditions on the relationships between most of the functional fruit traits in *S. dulcificum* suggests the possibility to conduct environment-specific traits selection and production. It further suggests the need to involve multi-environments or locations in evaluation and development of improved varieties of *S. dulcificum*.

Genetic gain is fundamental in plant breeding program and is a function of the heritability, the phenotypic variation and the selection intensity^81^. A joint analysis of the variability (variance) and the heritability (coefficient of repeatability) of the six functional fruit-traits in this study indicated that under similar selection pressure, a greater genetic gain may be achieved in fruit mass, seed mass and fruit width against a lower gain in the edible ratio. In fact, by nature, breeding for traits expressed in a ratio is considered more laborious than simple traits^77^, and this is well illustrated by the edible ratio of the miracle fruit that has an extremely low repeatability estimate (R = 0.015). On the contrary, this trait seemed to be more heritable in other fruit tree species (e.g. *A. trifoliate*^33^) where the repeatability estimate for edible ratio was as high as 0.98. This suggests a potential species-specific pattern in the heritability of this important trait. Fruit mass and seed mass had high heritability estimates which suggests that these traits could be easily improved in the miracle berry once suitable testing environments are selected. While those traits were also found to be highly heritable in *A. trifoliate*^33^, they were less heritable in *V. paradoxa*^34^ and *Allanblackia floribunda* Oliver^82^ among other indigenous fruit tree species.

Whether high heritability combined with large phenotypic variability can ensure consequent genetic gain, a good baseline breeding population is desired to accelerate genetic gain^83^. The development of a performing breeding population in tree breeding first relies on elite tree identification^84^. Findings from this study highlighted the existence of a diversity of breeding population pools in the miracle plant that can be tailored to specific breeding objectives. Fundamentally, it emerged that the Upper Guinea (UG) represented a source of promising parental lines that can be immediately exploited to initiate a breeding program in the miracle plant.

A core collection was developed, which in terms of size is approximately 20% of the size of the whole collection evaluated. This proportion is in line with the recommended size for a good core collection (10–30% of the initial collection)^85,86^. Historically, four parameters have been used to evaluate the internal quality of a core collection: the coincidence range (CR), the mean difference percentage (MD), the variance difference (VD) and the variable rate (VR)^60^. In this study, the core collection exhibited a coincidence rate CR that is by far higher than the threshold of CR > 80% required for a core collection to optimally represent an initial whole collection. While it was indicated that a well-constructed core collection should have no more than 20% of the traits deviating significantly from the whole collection, none of the traits in the core collection significantly deviated from the whole collection. The MD was also extremely low, suggesting that the core is a good representative of the whole set of accessions. Most important, the core collection encompassed individuals from all the sampled populations in this study, and all the clusters identified with a quasi-similar representativeness. Large VD and VR are preferred for a good collection^38^ as they illustrate how much diversity of the whole collection is maintained in the core collection. An exceptionally high variable rate (> 100%) and a VD of 46.95% were observed, which together suggests that further diversity can be obtained from the core collection compared to the whole collection. Combined, these characteristics suggest that the regional core collection compiled is robust enough to represent the whole collection and can consequently be considered as a reliable working sample in implementing ex-situ conservation measures and developing a breeding population for association study and further genomic selection implementation^87^ in the species. Practically, this core collection will ease and accelerate further evaluations (multi-location trials, genomic evaluation, metabolomic evaluation) and consequently shorten time to cultivar release. Besides, this core collection to be maintained as a living collection will help secure the reservoir of useful alleles the breeding process might need to tap in the future while favouring their co-evolution with environmental factors. An analysis of the core collection constitution indicated that all the three clusters identified in this study were represented in the Dahomey Gap zone whereas mainly one cluster was present in the Upper Guinea zone. This suggests the Dahomey Gap to be a centre of *S. dulcificum* diversity while providing an opportunity to properly elucidate phylogeographical relationships of the Dahomey Gap populations with the Upper Guinea, the Lower Guinea and the Congolian populations of the species.

In summary, this study evaluated the phenotypic variation in the miracle plant *S. dulcificum*, from its centre of origin, West Africa. A prominent effect of the provenance population was found on most of the phenotypic traits, with a better performance from the Upper Guinea forest accessions compared to their counterparts from the Dahomey Gap. Tree-traits were more variable than fruit-traits. Traits such as fruit mass, edible ratio and fruit width were highly correlated and exhibited high heritability estimates implying that simultaneous selection of the traits can be done. In addition, *S. dulcificum* individuals likely to serve as elite parental lines in the selection and development of high-yielding and pulp-dense cultivars were also identified. Finally, the successful development of a core collection of 41 accessions from the initial set of 203 accessions suggested the Dahomey Gap as a centre of diversity of the species. These findings pave the way for focused ex-situ conservation measures implementation as well as a cost-effective breeding program design for the species.

## Supplementary information

Supplementary Information

## Data Availability

All data generated or analysed during this study are included in this published article (and its Supplementary Information files).
