# Supplementary material for: Phenotypic variation, functional traits repeatability and core collection inference in Synsepalum dulcificum (Schumach & Thonn.) Daniell reveals the Dahomey Gap as a centre of diversity
Source: Sci Rep. 2020 Nov 11;10:19538. doi: 10.1038/s41598-020-76103-4 (PMC7658981; doi:10.1038/s41598-020-76103-4)
Supplement: Supplementary file 1 — Supplementary Information [file 41598_2020_76103_MOESM1_ESM.pdf]

Phenotypic variation, functional traits repeatability and core collection inference in *Synsepalum dulcificum* reveals the Dahomey Gap as a centre of diversity

Dèdéou A. Tchokponhoué[1,2*], Enoch G. Achigan-Dako[2], Sognigbé N'Danikou[2,5], Daniel Nyadanu[3], Rémi Kahane[4], Jacob Houéto[2], Nicodème V. Fassinou Hotegni[2], Alfred O. Odindo[1] & Julia Sibiya[1]

[1]School of Agricultural, Earth and Environmental Sciences, University of KwaZulu-Natal, Private Bag X01, Scottsville, 3209, Pietermaritzburg, South Africa.

[2]Laboratory of Genetics, Horticulture, and Seed Science (GBioS), School of Plant Sciences, University of Abomey-Calavi, BP 2549, Abomey-Calavi, Benin.

[3]Cocoa Research Institute of Ghana (CRIG), P. O. Box 8, Akim Tafo, Ghana

[4]Research Unit HortSys, Department Persyst, CIRAD, Campus de Baillarguet, 34398 Montpellier cedex 5, France

[5]World Vegetable Center, East and Southern Africa, PoBox 10 Duluti, Arusha, Tanzania

***Corresponding author:** Dèdéou A. Tchokponhoué

Email: dedeoutchokponhoue@gmail.com; **Tel: +27822536987**

Supplementary figures and tables

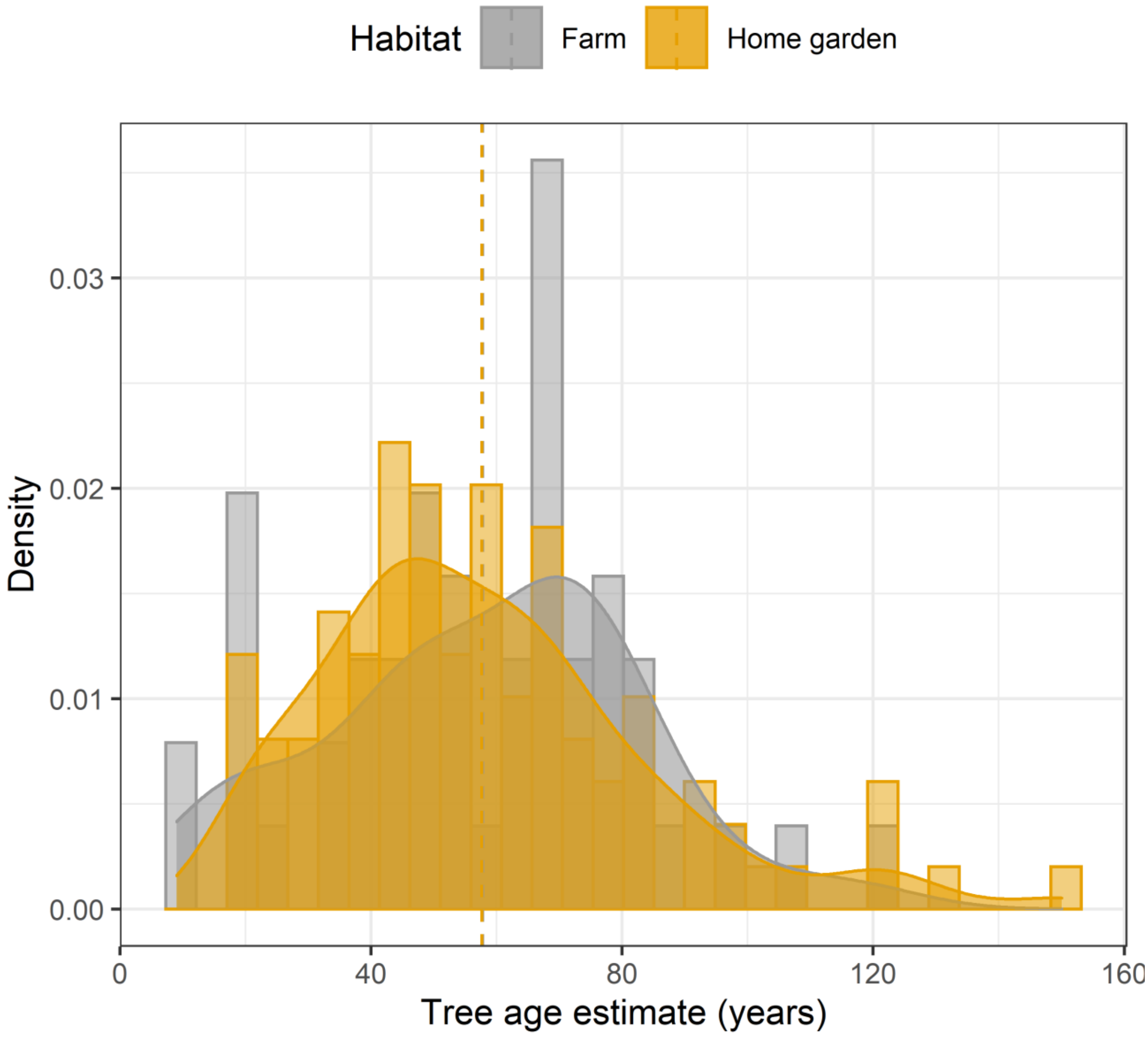

Figure S1. Variation of tree age estimate for *Synsepalum dulcificum* trees in home garden and on farm. Coloured vertical dashed lines (here almost confounded) represented mean age estimate following each habitat type.

Table S2. Spearman correlation between tree age estimate and tree- and fruit-traits.

**Tree-and fruit-traits**	**Tree age estimate**
Tree diameter at ground level (cm)	0.53^{***}
Tree height (m)	0.1^{ns}
Tree crown diameter (m)	0.3^{***}
Fruit length (mm)	-0.07^{ns}
Fruit width (mm)	-0.21**
Individual fruit mass (g)	-0.10^{ns}
Individual seed mass (g)	-0.03^{ns}
Edible ratio (%)	-0.09^{ns}
Fruit shape index	0.17*

***: $p < 0.001$, **: $p< 0.01$, *: $p < 0.05$, ns: Non-significant

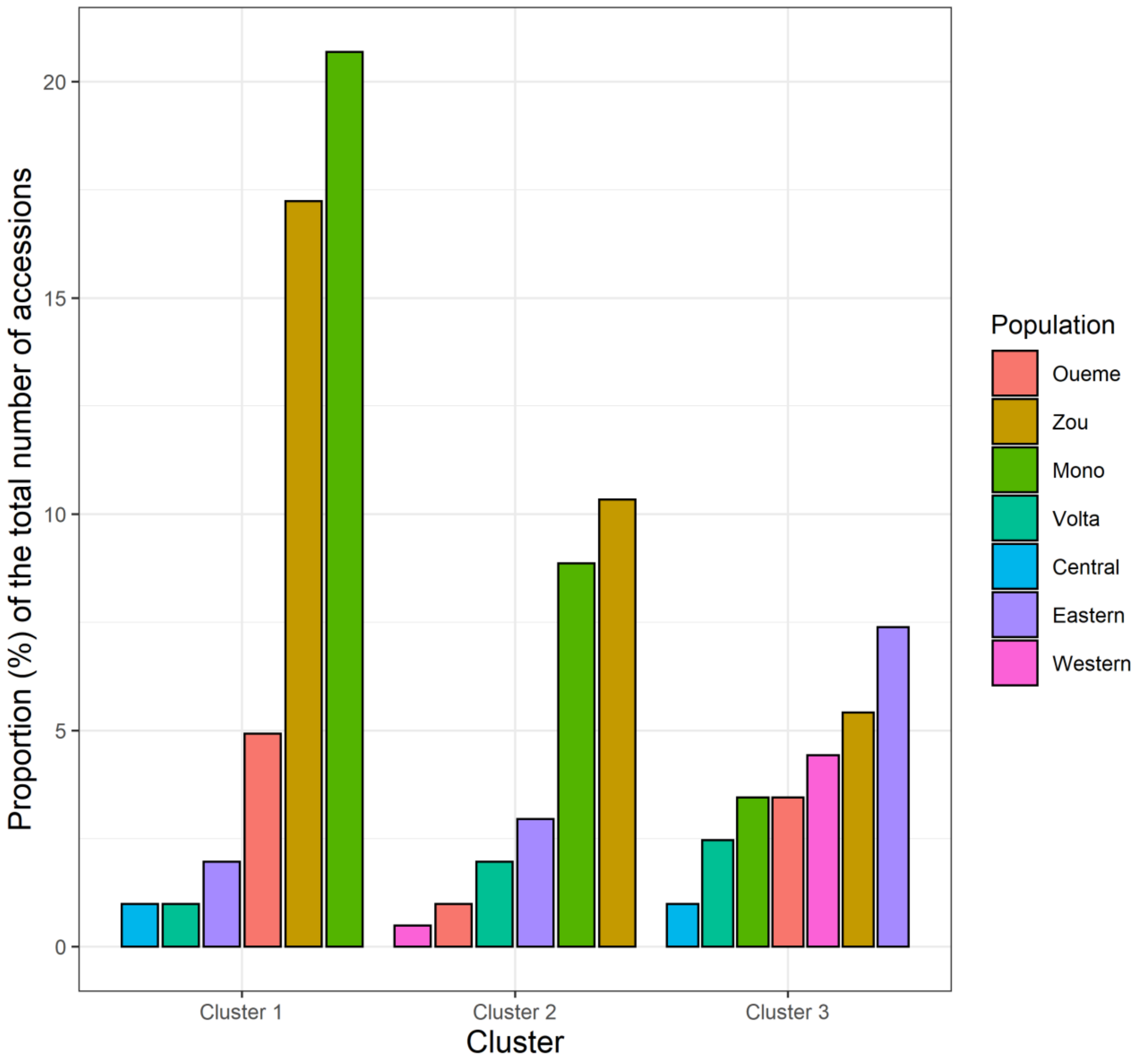

Figure S3. Relative contribution of *Synsepalum dulcificum* provenance populations to various clusters.
